# Advantages of Spray Drying over Freeze Drying: A Comparative Analysis of *Lonicera caerulea* L. Juice Powders—Matrix Diversity and Bioactive Response

**DOI:** 10.3390/molecules29153586

**Published:** 2024-07-30

**Authors:** Anna Michalska-Ciechanowska, Jessica Brzezowska, Paulina Nowicka, Karolina Tkacz, Igor Piotr Turkiewicz, Aleksandra Hendrysiak, Jan Oszmiański, Wilfried Andlauer

**Affiliations:** 1 Department of Fruit, Vegetable and Plant Nutraceutical Technology, Faculty of Biotechnology and Food Science, Wrocław University of Environmental and Life Sciences, 51-630 Wrocław, Poland; jessica.brzezowska@upwr.edu.pl (J.B.); paulina.nowicka@upwr.edu.pl (P.N.); karolina.tkacz@upwr.edu.pl (K.T.); igor.turkiewicz@upwr.edu.pl (I.P.T.); aleksandra.hendrysiak@upwr.edu.pl (A.H.); jan.oszmianski@upwr.edu.pl (J.O.); 2 Institute of Life Technologies, School of Engineering, University of Applied Sciences and Arts Western Switzerland (HES-SO Valais Wallis), Rue de l’Industrie 19, 1950 Sion, Switzerland

**Keywords:** haskap berry, powder, palatinose, inulin, trehalose, processing, antiglycation, anti-ageing properties

## Abstract

The study investigated the impact of *Lonicera caerulea* L. juice matrix modification and drying techniques on powder characteristics. The evaluation encompassed phenolics (514.7–4388.7 mg/100 g dry matter), iridoids (up to 337.5 mg/100 g dry matter), antioxidant and antiglycation capacity, as well as anti-ageing properties of powders produced using maltodextrin, inulin, trehalose, and palatinose with a pioneering role as a carrier. Spray drying proved to be competitive with freeze drying for powder quality. Carrier application influenced the fruit powder properties. Trehalose protected the phenolics in the juice extract products, whereas maltodextrin showed protective effect in the juice powders. The concentrations of iridoids were influenced by the matrix type and drying technique. Antiglycation capacity was more affected by the carrier type in juice powders than in extract products. However, with carrier addition, the latter showed approximately 12-fold higher selectivity for acetylcholinesterase than other samples. Understanding the interplay between matrix composition, drying techniques, and powder properties provides insights for the development of plant-based products with tailored attributes, including potential health-linked properties.

## 1. Introduction

In an era characterised by an ever-growing demand for natural and health-promoting food merchandise, the food industry has witnessed a paradigm shift towards the utilisation of plant-based ‘natural’ ingredients. Among numerous berry fruits, the haskap berry (*Lonicera caerulea* L.) stands out as a promising powerhouse of nutrients and phytochemicals, with the potential to diversify and enrich food products [1]. The haskap berry has captivated the attention of health-conscious consumers due to the presence of bioactives associated with specific health benefits, including antioxidant, anti-inflammatory [2], and most recently, antidiabetic properties [3]. As this berry is highly perishable, some attempts have been made to prolong its marketability through modern processing, such as fermentation [4] or drying [5]. The dried form of haskap berry fruit and extracts are increasingly available on the market for food and pharmaceutical applications. To date, the studies on the bioactives of the haskap berry have mostly been focused on the fruit, extract, and pomace, and the processing of this berry in terms of juicing was mostly considered [6,7]. However, the stabilisation of bioactives from this raw material by drying has scarcely been presented [8,9]. Furthermore, the conversion of juice, which is one of the main products of haskap berry processing, into a stable powder form has been little explored, and the contribution of the carrier and drying technique has been all the more neglected. As reviewed by Ueda et al. [10], food powders have been studied over the years from a variety of perspectives, starting with physical attributes and moving towards the evaluation of bioactive potential, which determines their exhibited biological and health-related properties. However, the processing aspect has been neglected, especially when considering drying techniques. Both conventional and more sophisticated drying methods are commonly used, depending on the matrix form (liquid, semi-liquid/solid, or solid) and the desired physico-chemical properties. Currently, spray drying (SD) and freeze drying (FD) are the most widely used in the food and pharmaceutical sectors to produce botanical powders. Each method has distinct advantages and drawbacks, making them the subject of scientific research into their influence on the plant matrix and improvement of the biological properties of powders. Another significant factor is the temperature and time of the process. In spray drying, a far-scalable process, a relatively high temperature may lead to the loss of nutrients and phytochemicals due to the thermal-induced degradation of these constituents and other chemical reactions. Conversely, in general, freeze drying maintains lower temperatures, minimising the loss of valuable components [11]. However, the freezing process can lead to the formation of ice, damaging the cell structure of the plant material. In addition, during the desorption phase after sublimation, water removal can be conducted at a higher temperature and for a longer time to achieve sufficient dehydration [11]. This process may affect the chemical composition of plant-based products in powder form, thereby altering their biological properties. Recently, it was demonstrated that the adjustment of the processing parameters of both drying techniques resulted in a better quality of plant powders, i.e., pea water [12], chokeberry [13], blueberry [14], and beetroot [15].

The carriers, until now considered non-reactive, were shown to affect the phenolic composition [13] and consequently the biological properties of plant-based powders [15]. From an industrial perspective, employing a carrier enhances the yield of powder production, thereby increasing encapsulation efficiency. Simultaneously, it constitutes a larger portion of the product formula (on a dry matter basis). This in turn cannot be neglected, especially when considering possible interactions with the food matrix [16].

Taking the above into consideration, the current study aimed for an evaluation of the effect of the *Lonicera caerulea* L. juice matrix composition, carrier type, and drying technique on the chemical (phenolics and iridoids) and biological properties, including the antiglycation and anti-ageing potential of powders. The matrix modification of fruit juice additionally strengthened by a carrier type used for drying may moderate the chemical properties of powders, which determine the biological potential of the final products. Furthermore, it was hypothesised that spray drying would enable the quality of the haskap berry juice and juice extract powder to be on par with, or potentially superior to, freeze drying.

## 2. Results and Discussion

### 2.1. Phenolics

Besides the initial composition of raw material, the modification of a fruit-based matrix by processing has an impact on the chemical and biological properties of the end products [9] that can be additionally altered by the type of processing additives, as in the case of plant powders - carriers [13]. In the present study, four major groups of phenolics were identified and quantified, namely, anthocyanins (4 compounds), phenolic acids (6), flavonols (12), and flavan-3-ols (2). The content of total phenolics in powders was linked to the presence of anthocyanins being a dominant group [1,17,18] that consisted of more than 60% of all identified components. 

In the case of controls (non-carrier-added samples), the products obtained from juice (J) had an approx. 11-fold-lower content of identified phenolics (Table 1 and Table 2), and therefore anthocyanins, than those produced from XAD extracts (XE), which was linked to the processing of the juice matrix [19]. When FD and SD were considered for XE controls, there was no statistically significant difference in total phenolics or anthocyanin content between the two techniques used (Table 1). 

Among the carriers used for production of juice powders, trehalose addition resulted in the highest content of anthocyanins when samples were FD, excluding peonidin-3-*O*-rutinoside. Palatinose addition caused the highest content of these constituents in products gained after SD. The anthocyanin content was linked to the presence of cyanidin-3-*O*-glucoside, which was present in all the powders analysed at more than 80% [2,20], followed by cyanidin-3,5-di-*O*-glucoside (approx. 13%), cyanidin- (2.8%), and peonidin-3-*O*-rutinoside (2.5%) [21]. In the case of SD products, the highest content of cyanidin-3-*O*-rutinoside was indicated for samples produced with the addition of palatinose; however, there were no statistically significant differences between the other carriers used. The peonidin-3-*O*-rutinoside was present in FD juice powders and only in samples to which maltodextrin was added before the spray-drying process. 

When considering the type of carrier, the addition of trehalose in XE led to the highest anthocyanin content for both drying techniques, while samples with added inulin exhibited the lowest anthocyanin levels. This affected the total phenolic content. A similar observation was made by Brzezowska et al. [22] in cranberry pomace extracts in which trehalose resulted in a higher content of anthocyanins in powders when compared to maltodextrin and inulin. Similarly to chokeberry powders, maltodextrin protected anthocyanins better than inulin [13]. This confirms that the preservation of specific anthocyanins from plant-based sources during drying depends on the type of carrier used [13] and can be attributed to variations in particles morphologies and drying rates during drying affected by the viscosity of the liquid feeds.

The second group of phenolics present in *Lonicera caerulea* L. powders consisted of phenolic acids (6), which represented, on average, 17% of all the identified and quantified components (Table 1). Similar to fresh haskap berries [1,18], 5-*O*-caffeoylquinic acid was a dominant phenolic acid in all the powders, accounting for more than 64% of phenolic acids present in the analysed products. Other phenolic acids present were di-*O*-caffeoylquinic acid, protocatechuic acid-4-glucoside and 1-*O*-caffeoylquinic acid, which accounted for an average of 12.4%, 11.1%, and 8.3% of all phenolic acids identified, respectively. The compounds detected at low levels were 3-*O*-caffeoylquinic acid and sinapic acid (below 3.6%). 

In the case of the non-carrier samples, like the anthocyanins, the SD of the XE resulted in a comparable content of the sum of phenolic acids to FD (no statistically significant differences, Tukey’s HSD, *p* < 0.05). Among the control samples, the modification of the juice matrix using resin (XAD-16) resulted in an 11-fold-higher content of phenolic acids in the powdered products. 

The addition of carriers lowered the content of phenolic acids, which was 5-times and 7-times lower in the case of juice and XE powders. Among the juice powders, the presence of trehalose resulted in the highest content of total phenolic acids, followed by maltodextrin, but only for SD samples. Generally, carrier type had a marginal effect on phenolic acid content in the powders. In the case of freeze-dried XE, the addition of trehalose before drying resulted in a higher content of phenolic acids, which was mainly linked to the content of 5-*O*-caffeoylquinic acid. The quantity of di-*O*-caffeoylquinic acid was higher in SD XE powders when compared to the other carriers applied. It can be concluded that SD can be considered as a method for encapsulating these components at levels comparable to FD. 

The content of flavonols was the highest in samples gained after the drying of XE (no carrier addition). In these samples, regardless of the drying technique used, twelve components were identified and quantified, whereas in the case of the FD juice control, six of them were detected (Table 2). This confirmed that the usage of polymeric resins to enable the concentration of flavonols was effective in the recovery of these plant secondary metabolites [19]. Among the drying techniques utilised in the production of XE powders, SD resulted in products with a significantly higher content of quercetin-*O*-rhamnoside-*O*-hexoside, kaempferol-3-*O*-hexoside, and quercetin-3-*O*-vicianoside. The remaining identified flavonols were found to be similarly stable during the application of both drying techniques. The study confirmed the thermal stability of the components belonging to this group of phenolics as previously indicated by Sharma et al. [23] and Michalska-Ciechanowska et al. [13]. 

When examining juice powders obtained through drying with specific carriers, only three components were identified and quantified in these samples due to the initial chemical composition of the liquid feed. There were no statistically significant differences observed when comparing the different carrier types (Table 2). The average content of flavonols after addition of carriers in XE products, regardless the type used, was 10-fold lower than in the control samples. The addition of carriers excluded the presence of certain flavonols, i.e., quercetin, kaempferol-rhamnosyl-di-*O*-glucoside, and quercetin-3-*O*-vicianoside in powders. 

In the study, two components that belong to flavan-3-ols were identified and quantified in *Lonicera caerulea* L. powders, i.e., (+)-catechin and (−)-epicatechin [1]. Both components were at similar levels in XE products gained without carrier addition (Table 2) as they were during processing, and thus matrix modification led to their quantitative alterations [9]. Their content in XAD extract products without the addition of carriers (controls) was 6-times higher when compared to products based on juice. Among the carriers used, the addition of palatinose to juice led to the obtainment of products with the lowest content of (+)-catechin, regardless of the drying technique used for juice dehydration. In the case of (−)-epicatechin, the addition of trehalose and palatinose to liquid feed prior to drying resulted in the absence of this component in the powder (Table 2). In the case of XE powders, both components were present in the powdered products; however, the content of (−)-epicatechin was higher, except in the trehalose-added samples gained after spray drying. 

### 2.2. Iridoids Content 

Iridoids have previously been identified and quantified in *Lonicera caerulea* L. berries, including their distribution in different parts of the fruit. It was also shown that pre-treatment methods and processing affect the iridoids content in haskap berry products, especially during juicing [24]. However, to date there has been no evidence of their presence in powdered berry products obtained by SD.

Three iridoids were identified and quantified in the powders analysed and their occurrence was linked to the product composition submitted to drying. In the case of the control (juice powders), a similar level of each identified iridoid was noted. The XE powders gained without carrier addition were differentiated by the content of 7-epi-loganin and pentosyl-loganin, which was linked to the drying technique used. In the case of the first mentioned compound, SD resulted in products with nearly double the content of this constituent, while the use of FD led to a higher content of pentosyl-loganin. This suggests potential differences in the thermal stability of these constituents. 

In FD juice products, loganic acid and 7-epi-loganin were present only in powders with maltodextrin, whereas the addition of inulin resulted in the occurrence of 7-epi-loganin (Table 2). No iridoids were identified and quantified in the SD juice powders, regardless of the type of carrier used, suggesting the influence of a relatively high-temperature-process-induced degradation of these constituents. The modification of the matrix composition (removal of ballast substances by XAD column, XE) resulted in the presence of iridoids in all the XE powdered products that were additionally modified by the type of carrier used for drying. However, in the case of trehalose, no pentosyl-loganin was indicated. Regardless of the drying technique used for the XAD extract, trehalose protected loganic acid and 7-epi-loganin to the highest extent among the carriers used. 

### 2.3. Antioxidant Capacity 

The antioxidant capacity of *Lonicera caerulea* L. powders was linked to the composition of the juice matrix submitted to drying. The highest antioxidant capacity was noted for control samples obtained from the XAD extract (after FD and SD), which was about 12-times higher for TEAC ABTS, and 10-fold higher for FRAP than the control samples produced from juice (FD) (Table 3). This was linked to the juice matrix modification [25,26]. Among non-carrier-added samples made from XE, freeze drying led to the obtainment of products with slightly higher TEAC ABTS and FRAP values of approx. 5% and 10%, respectively. The addition of carriers to juice for powder production resulted in lower TEAC ABTS and FRAP values of approx. 80%, and in the case of XE samples, the difference was approx. 90% when compared to the non-carrier-added sample. The ability to scavenge ABTS^•+^ radical cations and to reduce the Fe^3+^ to Fe^2+^ was approx. 84% lower in the case of juice products with carriers, when compared to XE products containing respective microencapsulation substances. In both matrices, addition of maltodextrin resulted in products with the lowest TEAC ABTS and FRAP values, regardless of the drying technique used for powder preparation (Table 3). The carriers, i.e., inulin, trehalose, and palatinose did not distinguish the samples based on the analysed antioxidant properties.

**Figure 1:**
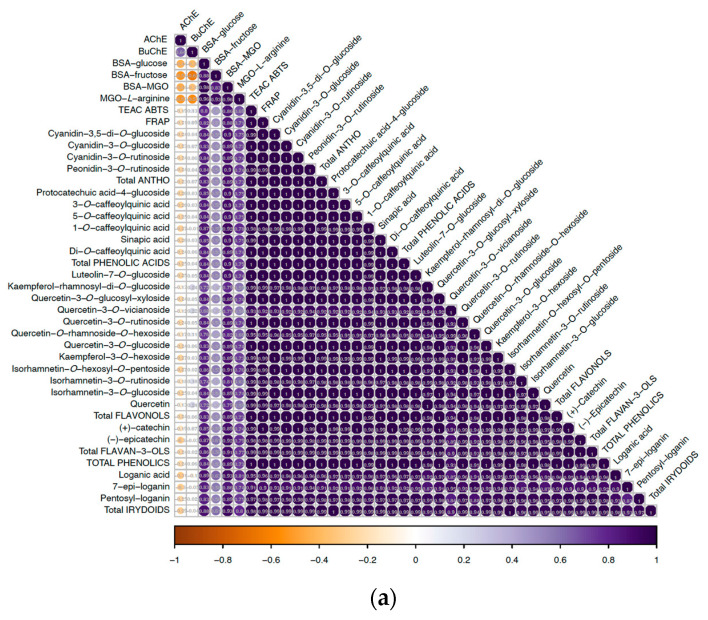

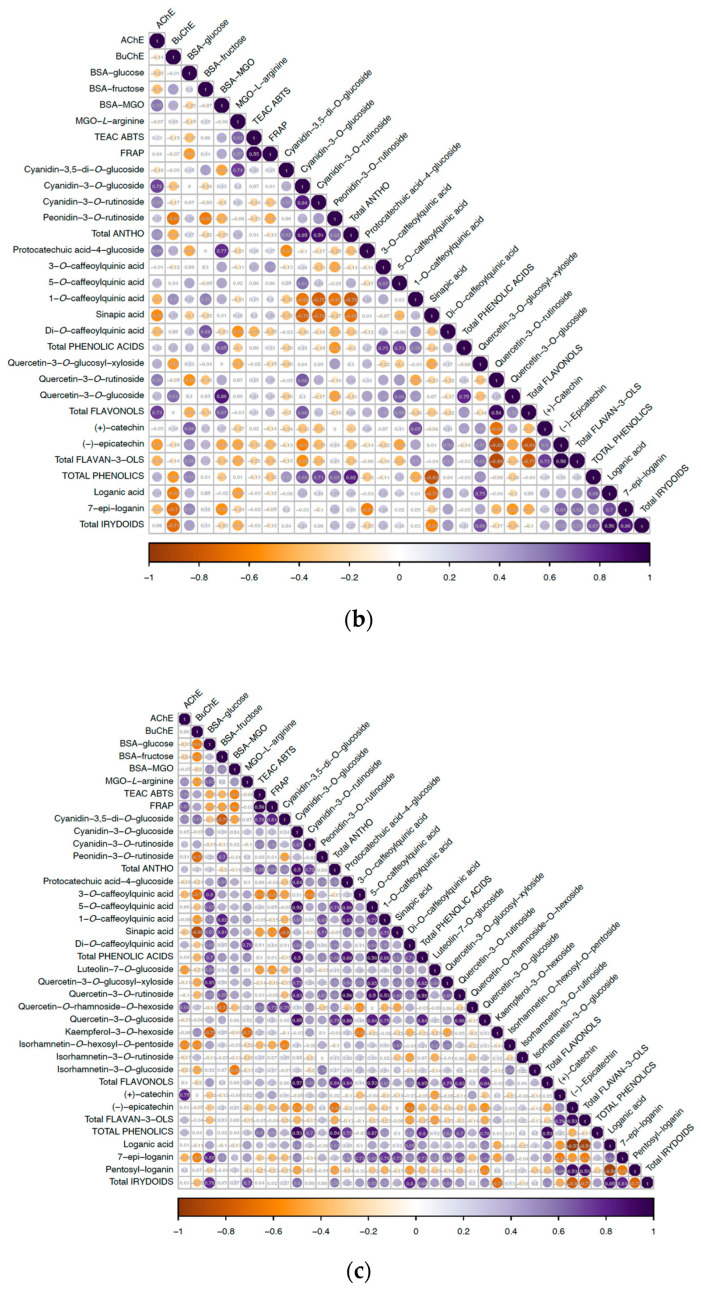
The correlation plots of (**a**) all data sets including control samples, juice, and XE powders with carriers; (**b**) juice powders with carriers only, excluding control samples; (**c**) XE powders only, excluding control samples. AChE—inhibition of acetylcholinesterase; BuChE—inhibition of butylcholinesterase; BSA—bovine serum albumin; MGO—methylglyoxal.

There was a strong correlation (above *r* = 0.95) between the antioxidant capacity measured by both in vitro methods and all identified phenolics (whole data set) (Figure 1a). It was associated with significantly higher values obtained for the control samples. In detail, no significant correlation between the juice powders, excluding the controls, was noticed between antioxidant capacity and specific phenolics, nor their groups (Figure 1b). When the XAD extract samples were considered, a strong positive correlation between total phenolic content and TEAC ABTS was indicated (*r* = 0.60), whereas in the case of FRAP a moderate linkage was observed (*r* = 0.54) (Figure 1c). 

Similarly, in *Lonicera caerulea* L. fruit analysed by Wojdyło et al. [1], a strong positive correlation between the total anthocyanins in juice powders with TEAC ABTS and FRAP was shown (*r* = 0.69 and *r* = 0.64, respectively), which confirmed the strong antioxidant potential of these components being dominant in haskap berries. Interestingly, there was no significant correlation between a dominant anthocyanin, i.e., cyanidin-3-*O*-glucoside; however, the greatest among the anthocyanins identified, with a significant correlation, was cyanidin-3,5-*O*-diglucoside, which was *r* = 0.78 and *r* = 0.81 for TEAC ABTS and FRAP, respectively. A similar observation was made by Fan et al. [17], who proved that this component was the major contributor to the reactive-oxygen-species (ROS)-scavenging activity in haskap berry extracts. This confirmed that even in the form of powdered products, this compound is active as an antioxidant. In the study, a positive correlation was also found between TEAC ABTS and FRAP, with one flavonol, i.e., quercetin-*O*-rhamnoside-*O*-hexoside (*r* = 0.62 and *r* = 0.72, respectively). This compound was present in XE powders, except products gained by spray drying with maltodextrin and inulin addition (Table 2), and in relatively small quantities; thus, its influence on antioxidant capacity is rather scarce. 

### 2.4. Antiglycation Capacity

The antiglycation effect of *Lonicera caerulea* L. powders without (controls) and with the addition of carriers was tested in different models to examine the inhibition of AGE formation under hyperglycemic conditions by powdered products toward different mechanisms of the glycation reaction in vitro (Table 3).

Both the BSA–glucose and BSA–fructose models that examine all the stages of the glycation of proteins were applied in the study as both carbohydrates are commonly present in the human diet [27]. In the BSA–glucose model, after 7 days of incubation, all the analysed powders exhibited the inhibitory effect towards AGEs, with the strongest impact of control samples (no carrier addition) obtained from juice and XAD extract products. There was no statistically significant difference (*p* < 0.05, Tukey’s HSD test) in the inhibitory effect of XE powders gained after FD and SD, whereas the control juice powders had approx. 20.4% lower ability to inhibit the formation of AGEs in this model. This can be ascribed to the initial composition, i.e., a higher content of phenolics in XAD extracts (Table 1 and Table 2). Additionally, the sugars present in *Lonicera caerulea* L. juice, mainly glucose and fructose, which on average are present at similar levels, dependent on the haskap berry variety [1], lowered the antiglycation capacity of the powders analysed. Among the juice powders, the products obtained without carrier addition had approx. 30% higher inhibitory ability than those obtained with their presence in the liquid feed. Among the carriers analysed, the application of maltodextrin led to the obtainment of freeze- and spray-dried products with a higher inhibitory effect than the rest of the carriers used. In the case of powders made from XAD extracts, the addition of carriers, on average, lowered the inhibitory effect by approx. 20.7% when compared to non-carrier-added products. There were no statistically significant differences between carrier-added samples and the drying technique applied (Table 3). Taking into account the results obtained for juice and XAD extract powders, it can be concluded that the type of carrier could affect the antiglycation capacity in samples with a lower content of phenolics, which confirms the dose-dependent effect of different molecules on the antiglycation capacity of plant-based products [28,29]. In the study, the single carriers used at the same concentrations, as applied for the powder production, exhibited an inhibitory effect, i.e., maltodextrin (2.7% ± 0.94), inulin (7.7% ± 1.9), and trehalose (9.9% ± 0.8), whereas palatinose increased AGE formation (−5.4% ± 2.8). Among the carriers, inulin and trehalose had a higher inhibitory effect in the BSA–glucose model than maltodextrin. It showed the trehalose probable inability to enter the glycation reaction [30]. This suggests that carriers added for plant-based powder production (in the case of *Lonicera caerulea* L. juice and XAD extracts: 30% (*w*/w)), especially due to their quantities used for liquid feed preparation (before drying) should be considered components that may affect the biological properties of the powdered products, including antiglycation capacity. However, further research linked to the effect of the carriers in the specific concentration used for drying should be evaluated in terms of the acceleration or deceleration of glycation. 

In the BSA–fructose model (Table 3), the inhibitory effect, measured as percentage of inhibition, was higher when compared to the BSA–glucose model, which confirmed that fructose was more reactive than glucose in the glycation reaction [29,31]. In this model system, the inhibitory effect of juice powders obtained without carrier addition was only approx. 5.5% lower when compared to products gained from XAD extract (control). Carrier application to juice lowered the antiglycation capacity of powders, on average, by approx. 25% when compared to control samples. Concerning the drying technique, among the carrier-added products, on average the SD samples (average 67.2%) had a higher inhibitory effect than the FD samples (62.8%) due to the lower value for samples obtained with palatinose addition. 

The addition of carriers into the XE resulted in only an approx. 4.2% decrease in antiglycation potential when compared to control samples, and no statistically significant differences were noted between the powders produced with the addition of the selected carriers and drying techniques applied. The inhibitory effect of AGE formation by carrier-added XE powders was approx. 40% higher when compared to their juice counterparts. 

In the study, the powders were tested for their inhibitory capacity regarding AGEs in the mid-stage of protein glycation [32]. In the BSA–MGO model, the effects of the control samples were similar to those obtained in the BSA–glucose/fructose models (XE powders had a greater ability to prevent glycation than juice-based products, and drying technique had no significant influence on these properties) (Table 3). The addition of carriers to juice and XAD extracts before drying lowered the inhibitory effect by, on average, approx. 48%. In the case of the juice products, the addition of inulin for the freeze drying of *Lonicera caerulea* L. powders resulted in the weakest inhibitory effect, whereas the addition of trehalose for spray drying led to the obtainment of products with the strongest inhibitory effect among the juice powders. On the other hand, in the case of the XE powders, maltodextrin led to the obtainment of products with a significantly greater ability to inhibit AGE formation. 

The results gained for the *L*-arginine-methylglyoxal model indicated a similar path as for the other models (Table 3); however, the inhibitory effect was significantly lower than that for BSA–glucose/fructose or MGO, which was linked to different mechanisms of the reaction [33]. In the study, the percentage of AGE inhibition did not exceed 10% for juice powders and 62% for XE powders. The addition of carriers to juice prior to drying significantly reduced the ability to inhibit the AGE formation of powders, and the presence of maltodextrin accelerated AGE generation, regardless of the drying technique used to produce the powders. Previously, the glycation reaction between BSA and maltodextrin induced by microwave and conventional heating was confirmed [34]; however, in the case of powders composed of maltodextrin (30%, *w*/w) or other carriers used in the study, the composition of powdered juice products, e.g., the presence of phenolics, may not be sufficient to prevent the reaction between arginine and methylglyoxal as other powder components, i.e., sugars may be more reactive. In view of the above, further studies should be carried out to evaluate the effect of carriers, including their type and concentration, with regard to their glycation potential, as plant powder products (including food supplements) usually contain carbohydrate-based carriers. The functional or health-related properties of such products cannot only be considered in terms of phytochemicals, but also in terms of the possible limitation of glycation reactions. 

Previously, it was proved that selected plant species, due to the presence and contents of phytochemicals, had an inhibitory effect towards AGE formation that was also linked to their antioxidant capacity, especially flavonoids [35], catechin [36], ferulic acid [37], *p*-coumaric, and chlorogenic acid [38]. Both activities of these natural components may differ in terms of the dependence on their structure and concentration in matrices, including interrelations and interactions between them [36]. Thus, the biological properties may differ due to the matrix alterations being additionally modified by processing. 

In the case of juice powders, excluding the controls, a highly positive correlation was only indicated between BSA–glucose and (+)-catechin among all the compounds identified (*r* = 0.66) (Figure 1b). Chen et al. [39] noticed that (+)-catechin was more effective on AGE inhibition than (−)-epicatechin, which was linked to its higher activity toward neutralisation of radicals, i.e., RO^•^, ^•^OH, and ^•^CHO. For the BSA–fructose model, a highly positive correlation was found between di-*O*-caffeoylquinic acid (*r* = 0.68). A strong positive correlation was also found for the BSA–MGO model and protocatechuic acid-4-glucoside (*r* = 0.77), together with total phenolic acids (*r* = 0.69). Also, a strong positive correlation was indicated for quercetin-3-*O*-glucoside (*r* = 0.86) and total flavonols (*r* = 0.67). Interestingly, the MGO–*L*-arginine results were highly positively correlated with TEAC ABTS (*r* = 0.63) and FRAP values (*r* = 0.57). When the particular phenolics were concerned, there was a strong positive correlation in this antiglycation model and cyanidin-3,5-di-*O*-glucoside (*r* = 0.74) (Figure 1b), thus the compound being highly responsible for antioxidant capacity.

In the case of XAD extract powders, as the matrix were modified in terms of chemical composition, a highly positive correlation between BSA–glucose and 4 out of 6 identified phenolic acids, i.e., 3-*O*-caffeoylquinic (*r* = 0.80), 5-*O*-caffeoylquinic and sinapic (*r* = 0.67), as well as di-*O*-caffeoylquinic acid (*r* = 0.62) was indicated giving the correlation coefficient between total phenolic acids equal to *r* = 0.7. A moderate positive correlation was established between this parameter and 1-*O*-caffeoylquinic acid (*r* = 0.53). Among flavonols, a highly positive correlation between this variable and quercetin-3-glucosyl-xyloside (*r* = 0.85), luteolin-7-*O*-glucoside (*r* = 0.64) and with one iridoid, i.e., 7-epi-loganin (*r* = 0.82) and thus total iridoid content (*r* = 0.78) was indicated. Previously, it was reported that selected iridoids may have antiglycation capacity [40]. 

In the BSA–fructose model, among all anthocyanins identified, there was a strong high correlation with peonidin-3-*O*-rutinoside (*r* = 0.70). In the case of phenolic acids, a strong positive correlation was noticed for: protocatechuic acid-4-glucoside (*r* = 0.61), 1-*O*-caffeoylquinic (*r* = 0.82), and sinapic acid (*r* = 0.81). Among flavonols, there was a strong positive correlation between BSA–fructose and quercetin-3-*O*-rutinoside (*r* = 0.69). When the iridoids were concerned, a weak positive correlation between BSA–fructose and 7-epi-loganin (*r* = 0.51) was indicated. In the case of MGO–*L*-arginine, there was a strong positive correlation between antiglycative potential in this model and di-*O*-caffeoylquinic acid (*r* = 0.78) and total iridoids (*r* = 0.7). 

In this view, *Lonicera caerulea* L. juice and XAD extract powders may be considered products with antiglycation potential. Juice matrix modification, and thus the chemical composition, moderated the antiglycation capacity of the analysed powders. The particular phenolics present in the juice and XAD extract powders played a different role in the inhibition of AGE formation in the analysed models. Anthocyanins, being a dominant group of phenolics, did not play a significant role in the prevention of the glycation process. Wang et al. [36] indicated that selected compounds from this group may be considered as potential MGO scavengers; however, it was suggested that anthocyanins were less effective than anthocyanin–procyanidin mixes. Following Wang et al. [36], it was confirmed that an estimation of the total anthocyanin content in berry juice or extracts may not accurately represent their biological effects. It can be concluded that some phenolics may prevent better glycation, dependent on the stage [28], and in such complicated matrices as haskap berry juice and XAD extracts, its evaluation should be made for end-products and powders, as processing may lead to the changes in phenolic presence and content.

### 2.5. Anticholinesterase Capacity

Given the importance of finding effective natural inhibitors of acetylcholinesterase (AChE) and butylcholinesterase (BuChE) for the treatment or amelioration of age-related disorders, the haskap berry powders were tested for their anticholinergic capacity. The IC_50_ varied from 11.7 up to 26.9 mg of sample per mL of enzyme for AChE and from 12.8 up to 69.0 mg/mL for BuChE, with statistically significant differences (*p* < 0.05, Tukey’s HSD test) between powders obtained using different processing criteria (matrix modification, carrier addition and type, and drying technique). 

Considerably wider fluctuations in inhibition levels between samples were observed for anti-BuChE than for anti-AChE capacity. Going into the details, despite such substantial differences in the content of phenolics and iridoids present in the powder matrices studied (Table 1 and Table 2), no tendential linkage was observed between the processing used (matrix type, carrier type, and drying method) and the anti-AChE capacity. All products showed relatively comparable anti-AChE capacity, with IC_50_ values ranging from 11.7 to 26.9 mg/mL. Among the juice powders obtained by FD and SD, only the lyophilised sample with added inulin displayed a weaker ability to inhibit AChE, while the rest of the products in this set revealed no statistically significant differences, regardless of carrier application and type, as well as drying technique. Therefore, it can be assumed that the processing applied did not moderate the anti-acetylcholinesterase capacity to a significant extent. 

On the other hand, a very clear parallel was observed in the case of the ability to inhibit butylcholinesterase by powders produced from XE, but only those with the carrier application. For all of them, the IC_50_ value was not lower than 56 mg/mL, which is puzzling, especially considering that the corresponding variants based on juices (matrix characterised by a lower content of phenolics and iridoids, and therefore lower antioxidant and antiglycation potential) showed greater anti-BuChE capacity. A possible explanation for this may be that the encapsulation effectiveness of the bioactives present in a simplified matrix of XE (no ballast components such as sugars or organic acids being removed during the purification process), and therefore their higher affinity for the shell material [41], hindered their release into the environment during extraction prior to analysis, and thus attenuated the ability of the powders to inhibit butylcholinesterase capacity. The migration of the core from the microcapsule matrix may also be determined by its spatial distribution within the structure and may therefore be responsible for the specific effects of the bioactive compounds released into the environment during the extraction process [41,42]. The differences may also be due to specific docking mechanisms resulting from the inherent structure of each enzyme, namely the difference in the gorge space of AChE and BuChE, and thus the different ability to accommodate molecules extracted from powders within it [43]. 

Although no clear drying-dependent patterns were observed in the present study, Somjai et al. [44] showed that drying as well as long-term storage can enhance, among others, cholinesterase inhibition activities, which was ascribed to the formation of a sugar–protein conjugate via the Maillard reaction and/or glycosylation. The results of this study have shed new light on those transformations that are generally considered undesirable but which, under controlled conditions, can improve the bioactive properties of foods, including anti-ageing properties. In addition, Turkiewicz et al. [45] noted a highly positive correlation between hydroxymethyl-*L*-furfural (HMF), being a major indicator of the Maillard reaction and caramelisation, and the ability to inhibit cholinesterase enzymes for Japanese quince powdered XAD extract obtained by different drying techniques. What is more, regarding anti-AChE capacity, no differences were found between freeze- and spray-dried samples; however, a higher temperature during vacuum drying improved this ability. Such observations can support our study, which did not reveal a strict pattern when considering drying methods. Nevertheless, it should be noted that the techniques chosen have previously been shown to have a comparable effect on the quality of fruit powders, with the lowest content of hydroxymethyl-*L*-furfural [13], and thus process contaminants, which may be responsible for anti-ageing properties. Therefore, further studies using other drying parameters are required to fully recognise the processing–anti-ageing interrelation, especially when considering multi-compositional matrices such as fruit powders.

When considering the carrier type applied for powder production, the only difference was noted for inulin-supplemented products among freeze-dried juice and spray-dried XE sample sets, for which the weakest ability to inhibit AChE capacity was observed (Table 3). However, this did not coincide with the results for AChE inhibition. The explanation for this can be sought in the so far unrecognised interactions between the matrix and this carrier exposed to the selected processing. To date, modified inulin has been suggested as a potentially attractive emulsifier for the design of anti-ageing formulations [46], but there is a paucity of information on its efficacy as an encapsulating agent in terms of further product neuroprotective potential, particularly in the food field. Moreover, to the best of our knowledge, there is no literature presenting the carrier-dependent effects of fruit powders on anti-ageing properties; therefore, the results presented provide valuable insights, especially for the design of instant foods with targeted biological properties, including anticholinesterase capacity. Hence, dedicated model studies are needed to better understand the effect of carrier application on anti-ageing properties as a function of matrix complexity. 

**Figure 2:**
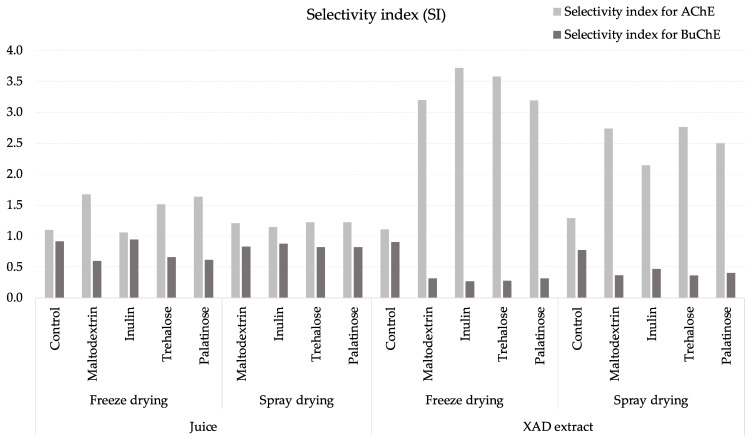
Selectivity index (SI) calculated for AChE (BuChE IC_50_/AChE IC_50_) and BuChE (AChE IC_50_/BuChE IC_50_) of haskap berry powders. AChE—inhibition of acetylcholinesterase; BuChE—inhibition of butylcholinesterase.

To estimate the relative potency for the inhibition of acetylcholinesterase (BuChE IC_50_/AChE IC_50_ ratio) and butylcholinesterase (AChE IC_50_/BuChE IC_50_ ratio), selectivity indices were calculated (Figure 2).

The powders produced from XE with carrier application displayed much greater selectivity towards AChE (approx. 12-times higher) than the rest of the samples. On the other hand, spray-dried products from haskap berry juice seemed to have dual AChE–BuChE inhibitory activity with comparable selectivity. Nevertheless, all powders exhibited greater selectivity for AChE than for BuChE.

When analysing the powders as a collective set, there was a moderate positive correlation between anti-AChE and anti-BuChE capacity (*r* = 0.64) (Figure 1a). This could point to the fact that the phytochemicals present in the powders constitute a combination of both anti-AChE and anti-BuChE agents, or that these bioactives have dual potency to inhibit cholinesterase enzymes at the same time [47]. Therefore, regarding the influence of specific phenolic and iridoid compounds and their groups, the powders were considered in a split between the two matrices (J powders without controls; XE powders without controls) in order to reveal relationships that could be masked by an excessively diverse set of samples in terms of chemical composition (juice; XAD extracts), but without control inclusion. In general, the consistent linkage for both matrices was the high negative correlation between anti-BuChE and peonidin-3-*O*-rutinoside, which was *r* = −0.65 and *r* = −0.72 for J and XE powders, respectively (Figure 1b,c), which was not found for anti-AChE capacity. A similar observation was made for 7-epi-loganin, for which a high negative correlation was found between its content and anti-BuChE capacity in XE powders (*r* = −0.60) and J products (*r* = −0.70). Although the AChE inhibitory potential of iridoids has been demonstrated [48,49], the lack of correlation between iridoids and anticholinesterase capacity in the present study does not corroborate this. One possible reason for this may be that the binding energy requirements vary according to the structure of the molecule, and therefore other components may compete with them for full docking at the enzyme active site [50]. 

An interesting observation was made for (+)-catechin, namely a strong positive correlation was reported with anti-AChE (*r* = 0.79) when considering XE powders; however, this was not confirmed for products based on the juice matrix (*r* = −0.05) (Figure 1b,c). Additionally, when looking only at juice powders, a highly positive relationship was reported between anti-AChE and the sum of flavonols (*r* = 0.73), as well as cyanidin-3-*O*-glucoside (*r* = 0.72) (Figure 1b). Flavonols, together with phenolic acids, have previously been postulated to have greater potency as neuroprotectants than, for example, flavan-3-ols [51,52]. Moreover, Wu et al. [53] found that specific phenolics, including quercetin, were responsible for acetylcholinesterase inhibition. On the other hand, for XE powders, more strong negative dependencies were found, e.g., between anti-BuChE capacity and sinapic acid (*r* = −0.86) or 3-*O*-caffeoylquinic acid (*r* = −0.70) (Figure 1c). 

Inconsistency in the interplay of individual compounds or groups of compounds on enzyme activity, or their contrasting behaviour, may be due to the different ability of the chemical structure (glycosides/aglycones, presence of 3’-hydroxyl groups, etc.) of a given compound to suppress cholinesterase enzymes [51,54]. Moreover, this issue has previously been raised by Capuano et al. [55], where it was pointed out that the contrasted response of a specific compound may be influenced by the surrounding matrix in which it is present, and may therefore affect the chemical reactivity, and consequently, the inherent biological activity displayed by the product. To this end, it is of paramount importance to conduct more sophisticated and comprehensive studies using advanced model systems, not only to validate these findings, but also to provide deeper insights and a robust platform for future scientific progress in the field of food powders. 

## 3. Materials and Methods 

### 3.1. Materials

The *Lonicera caerulea* L. fruits (approx. 60 kg) were mechanically collected at commercial maturity from ‘Gospodarstwo Sadownicze Mikołaj Marczak‘ (Białężyn, Poland). Fruits were washed and frozen. As carriers, maltodextrin (Pepees S.A., Łomża, Poland), inulin (Beneo-Orafti, Oreye, Belgium), trehalose (Hayashibara Co., Okayama, Japan), and palatinose (PST-N, Beneo-Palatinit GmbH, Mannheim, Germany) were used. 

### 3.2. Methods

#### 3.2.1. Juicing and Preparation of Sugar Free XAD Extracts 

The thawed fruits were crushed in Thermomix (Wuppertal, Vorwerk, Germany) at 50 °C followed by enzymation (Pectinex BE XXL; 2 mL of 1:10 (*v*/*v*) diluted enzyme for each 2 kg of fruit) for 1 h. The pulp was pressed (hydraulic press) to produce the juice (10.2 °Brix). The juice (J) obtained was divided into two equal parts: one part was used to prepare the XAD extract (XE) (10.4 °Brix) by using resin (Amberlite XAD-16, Brenntag, Poland) according to Kammerer et al. [19] to recover the selected phenolics. The second part was directly used for powder production.

##### Basic Chemical Properties of *Lonicera caerulea* L. Fruit and Its Fractions 

The dry matter (dm) was calculated according to Figiel et al. [56]. The titratable acidity (TA) was determined with a TitroLine 5000 (Xylem Analytics GmbH, Weilheim, Germany) with 0.1 mol/L NaOH to pH 8.1 and was expressed as mg malic acid/100 g of sample (PN-EN 12145:2000). Ash and ascorbic acid content was analysed according to norm PN-90/A-75101/08 and PN-A-04019 [57]. Pectin content (g/100 g) was measured according to the Morris method [57]. All measurements were made in duplicate (*n* = 2); results are presented in Table 4.

#### 3.2.2. Drying of Juice and XAD Extracts 

Juice (J) and XAD extracts (XE) were divided into equal portions and mixed with each carrier at 30% (*w*/w) (the J/XE:carrier solids ratio—1:3). Obtained liquid feed compositions were submitted to freeze drying (FD) (FreeZone, Labconco Corp., Kansas City, MO, USA; 24 h, 65 Pa, temperature of drying chamber and heating plate: −60 °C/25 °C) and spray drying (SD) (Mini Spray dryer B-290 Advanced, Büchi, Flawil, Switzerland; inlet temperature: 130 °C; outlet temperature: 72–84 °C; volume flow for sample with maltodextrin, inulin, and trehalose: 38 m^3^/h; for palatinose: 26 m^3^/h; feed flow: 4 mL/min). The powders obtained were vacuum-packed (MC 2006; Tepro SA, Koszalin, Poland) and stored at −20 °C until analysed. 

#### 3.2.3. Chemical Composition and in Vitro Biological Properties of Powders

##### Quantification of Phenolics and Iridoids 

Powders produced from J and XE were extracted with 30% aqueous methanol (*v*/*v*). The extraction was performed in duplicate (*n* = 2) by ultrasonication with shaking for 15 min. Samples were left for 24 h at 4 °C and then sonicated again (15 min). Next, the samples were centrifuged at 19,000× *g* for 10 min, and the obtained supernatants were filtered using a hydrophilic PTFE 0.20 μm membrane (Millex Samplicity Filter, Merck, Darmstadt, Germany) before analysis. The content of phenolics and iridoids was determined by means of ultra-performance liquid chromatography–photodiode array detector–quadrupole time-of-flight mass spectrometry (UPLC-PDA-QTof-MS/MS) (*n* = 2) [58]. The results were expressed as mg per 100 g dry matter (dm).

##### Antioxidant Capacity 

The in vitro antioxidant capacity of *Lonicera caerulea* L. powders was assessed by the TEAC ABTS^•+^ and FRAP assays using a Synergy H1 spectrophotometer (BioTek Instruments Inc., USA) [13]. Quantification was conducted in terms of millimoles of Trolox equivalent per 100 grams dm (*n* = 2).

##### Antiglycation Assays in BSA–Glucose/Fructose, BSA–Methylglyoxal and L-Arginine-Methylglyoxal Model 

The antiglycation capacity of *Lonicera caerulea* L. powders was analysed using 3 different models established according to Wang et al. [59] and Brzezowska et al. [15] with a modified extraction procedure, namely the omission of solvent evaporation and redissolution in phosphate buffer (procedure simplification). To this end, *Lonicera caerulea* L. powders were directly extracted (*n* = 2) with 30% aqueous methanol (*v*/*v*) (15 min of sonication, 24 h at 4 °C, 15 min of sonication, centrifugation at 19,000 × g, 20 °C using MPW-251, MPW Med. Instruments, Warszawa, Poland). Supernatants were tested for the initial (bovine serum albumin (BSA)–glucose and BSA–fructose model) and middle stage (BSA–methylglyoxal) of glycation as well as to track major and specific AGE formation (*L*-arginine–MGO model). The positive control was aminoguanidine (AG; 10 mM, final concentration). The measurements were performed by a Synergy H1 spectrophotometer (BioTek Instruments Inc., USA) at excitation and emission wavelengths of λ = 340 and λ = 420 nm for BSA–glucose and BSA–fructose, and λ = 340 and λ = 380 nm for the BSA–MGO and *L*-arginine–MGO models. The results (*n* = 2) were expressed as the % of the inhibition of AGE formation and were calculated using the Equation (1):

% inhibition = (1 − (Fl_powder_/Fl_control)_) × 100 (1)

where Fl_powder_ = fluorescence intensity of *Lonicera caerulea* L. powders and Fl_control_ = fluorescence intensity of control

##### Inhibition of Acetylcholinesterase (AChE) and Butylcholinesterase (BuChE) 

The extracts prepared for the determination of phenolics and iridoids were used for the evaluation of anticholinergic activity based on Ellman’s method by AChE and BuChE inhibition activity as reported by Wojdyło et al. [58] and modified by Tkacz et al. [52]. A negative test was performed without adding the enzyme, while physostigmine was used as reference. The obtained results were expressed as IC_50_ (mg of sample/mL of enzyme). The measurements (*n* = 2) were taken using a Synergy™ H1 microplate reader (BioTek; Winooski, VT, USA). The selectivity index (SI) was calculated for acetylcholinesterase as BuChE IC_50_ values/AChE IC_50_ values, and for butylcholinesterase as AChE IC_50_ values/BuChE IC_50_ values [50]. 

#### 3.2.4. Statistical Analysis

The statistical analysis was conducted using Statistica 13.1 (StatSoft, Kraków, Poland). To determine significant differences (*p* < 0.05) among the average values, an analysis of variance (ANOVA) was conducted followed by Tukey’s (HSD) post-hoc test. The Pearson correlation coefficient (*r*) was computed to explore the relationships among the chosen variables, and the correlation matrix was prepared using the ‘corrplot’ R package.

## 4. Conclusions 

In this study, the production of powders from *Lonicera caerulea* L. juice and XAD extracts by FD and SD was successfully performed using maltodextrin, trehalose, inulin, and palatinose (pioneering role as a carrier). In the powders, four groups of phenolics, namely anthocyanins (dominant group), phenolic acids, flavonols, and flavan-3-ols, were identified and quantified. Moreover, iridoids were identified for the first time in *Lonicera caerulea* L. powders. Their presence was affected by matrix modification, and carriers applied for the removal of the ballast substance by XAD resin from juice resulted not only in an increase in quantity, which was reflected in antioxidant capacity, but also in compositional changes within this fruit matrix. In general, in haskap berry products, maltodextrin better preserved phenolics in juice powders, whereas trehalose was more effective in preserving anthocyanins in XE products. Conversely, inulin resulted in samples with the lowest quantity of these compounds. The impact of both, the addition and carrier type, was more evident in juice powders compared to XE regarding their antiglycation capacity. Haskap berry powders showed significant anticholinergic potential, with varying levels of inhibition for acetylcholinesterase and butylcholinesterase depending on the processing methods. Matrix modification, rather than carrier type or drying technique, had a much greater impact on anti-ageing properties. In turn, the carrier-added powders showed markedly higher selectivity for AChE compared to BuChE, especially those derived from XE, suggesting that the encapsulation process may influence the release and activity of bioactive compounds. It was supposed that the differences in compounds’ chemical structure and how they interact with surrounding constituents in the matrix can influence their action and effectiveness, and thus the biological activity they exert. 

These findings highlight the potential of using different matrices of powdered haskap berries as natural inhibitors of glycation and cholinesterase enzymes, which could be beneficial in the development of treatments for age-related disorders. However, further research is needed to fully comprehend the role of specific processing approaches on their bioactive properties. According to the findings of this study, spray drying can be regarded as a competitive drying technique compared to freeze drying in relation to the chemical and biological properties of haskap berry powders. However, depending on the matrix utilised, the carriers can modulate these properties differently. Therefore, further investigation is warranted regarding their role in interactions and health-related effects within appropriate model systems.

## Figures and Tables

**Table 1:** The content of anthocyanins and phenolic acids in *Lonicera caerulea* L. powders (mg/100 g dry matter).

Sample	Drying Technique	Carrier	Anthocyanins				Phenolic Acids					
			Cyanidin-3-*O*-Glucoside	Cyanidin-3,5-Di-*O*-Glucoside	Cyanidin-3-*O*-Rutinoside	Peonidin-3-*O*-Rutinoside	5-*O*-Caffeoyl-Quinic Acid	Di-*O*-Caffeoylquinic Acid	Protocatechuic Acid-4-Glucoside	1-*O*-Caffeoyl-Qunic Acid	3-*O*-Caffeoyl-Quinic Acid	Sinapic Acid
J	FD	-	1442.39 ± 128.26 ^A^	221.59 ± 14.32 ^A^	50.12 ± 6.98 ^A^	47.92 ± 6.89 ^A^	290.50 ± 14.33 ^A^	60.56 ± 4.60 ^A^	52.00 ± 2.42 ^A^	48.14 ± 2.79 ^A^	10.28 ± 0.95 ^A^	4.42 ± 0.44 ^A^
XE	FD	-	16837.80 ± 1689.78 ^B^	2480.74 ± 189.93 ^B^	534.63 ± 109.58 ^B^	509.84 ± 31.05 ^B^	3478.01 ± 5.18 ^B^	691.03 ± 1.36 ^B^	592.28 ± 25.64 ^B^	314.18 ± 33.79 ^B^	128.94 ± 8.17 ^B^	48.74 ± 0.39 ^B^
XE	SD	-	15783.86 ± 30.84 ^B^	2535.96 ± 66.95 ^B^	504.21 ± 39.80 ^B^	543.71 ± 10.41 ^B^	3297.20 ± 106.05 ^B^	680.08 ± 33.62 ^B^	580.68 ± 32.36 ^B^	370.32 ± 9.04 ^B^	121.44 ± 1.88 ^B^	49.64 ± 1.37 ^B^
J	FD	M	303.81 ± 8.37 ^b^	42.46 ± 4.51 ^a^	11.30 ± 0.34 ^a^	12.01 ± 1.69 ^a^	56.68 ± 1.50 ^a^	11.43 ± 0.45 ^a^	9.12 ± 1.51 ^a^	7.33 ± 0.02 ^a^	2.77 ± 0.10 ^a^	0.60 ± 0.02 ^a^
		I	284.35 ± 4.56 ^ab^	48.38 ± 2.99 ^a^	9.15 ± 2.13 ^a^	9.74 ± 0.58 ^a^	54.75 ± 0.77 ^a^	10.84 ± 0.76 ^a^	7.53 ± 0.14 ^a^	7.41 ± 0.77 ^a^	2.59 ± 0.07 ^a^	0.82 ± 0.05 ^a^
		T	307.65 ± 6.27 ^b^	52.55 ± 14.10 ^a^	13.18 ± 0.13 ^a^	12.79 ± 2.35 ^a^	56.42 ± 1.58 ^a^	9.59 ± 1.86 ^a^	9.17 ± 2.66 ^a^	6.77 ± 0.49 ^a^	2.42 ± 0.26 ^a^	0.69 ± 0.02 ^a^
		P	298.41 ± 5.65 ^ab^	36.99 ± 3.63 ^a^	10.61 ± 1.30 ^a^	13.37 ± 0.64 ^a^	54.71 ± 2.11 ^a^	9.73 ± 0.36 ^a^	9.52 ± 1.07 ^a^	6.47 ± 0.73 ^a^	2.53 ± 0.23 ^a^	0.79 ± 0.29 ^a^
	SD	M	291.11 ± 9.82 ^ab^	38.56 ± 7.38 ^a^	11.28 ± 1.12 ^a^	11.83 ± 0.36 ^a^	55.56 ± 1.61 ^a^	10.85 ± 0.41 ^a^	9.34 ± 1.03 ^a^	7.31 ± 0.07 ^a^	2.33 ± 0.43 ^a^	0.73 ± 0.05 ^a^
		I	278.73 ± 2.75 ^a^	39.80 ± 4.42 ^a^	8.23 ± 0.16 ^a^	<LOD	52.94 ± 2.07 ^a^	11.01 ± 0.54 ^a^	10.45 ± 1.15 ^a^	8.15 ± 0.24 ^a^	2.47 ± 0.27 ^a^	0.81 ± 0.03 ^a^
		T	294.15 ± 5.69 ^ab^	38.55 ± 5.98 ^a^	8.61 ± 0.83 ^a^	<LOD	58.52 ± 1.26 ^a^	10.48 ± 1.76 ^a^	9.48 ± 0.83 ^a^	8.11 ± 1.04 ^a^	2.99 ± 0.88 ^a^	0.79 ± 0.02 ^a^
		P	302.55 ± 0.15 ^ab^	49.17 ± 7.62 ^a^	12.31 ± 3.42 ^a^	<LOD	56.08 ± 0.83 ^a^	11.38 ± 1.43 ^a^	8.85 ± 0.52 ^a^	6.99 ± 0.38 ^a^	2.66 ± 0.37 ^a^	0.68 ± 0.20 ^a^
XE	FD	M	2154.08 ± 43.47 *^ab^*	382.10 ± 17.10 *^ab^*	75.94 ± 3.34 *^a^*	72.62 ± 10.12 *^a^*	503.64 ± 15.38 *^ab^*	98.37 ± 1.86 *^ab^*	89.44 ± 1.51 *^ab^*	64.44 ± 0.90 *^a^*	19.60 ± 0.12 *^a^*	7.46 ± 0.06 *^a^*
		I	2074.61 ± 30.09 *^a^*	375.65 ± 0.44 *^ab^*	69.10 ± 4.19 *^a^*	77.62 ± 0.18 *^a^*	490.00 ± 5.92 *^a^*	93.72 ± 1.46 *^ab^*	85.90 ± 0.87 *^a^*	62.75 ± 4.06 *^a^*	20.29 ± 0.77 *^a^*	7.59 ± 1.22 *^a^*
		T	2256.54 ± 25.87 *^b^*	410.92 ± 23.51 *^b^*	77.81 ± 4.09 *^a^*	78.91 ± 7.01 *^a^*	525.74 ± 0.43 *^b^*	95.72 ± 0.97 *^ab^*	93.01 ± 0.46 *^ab^*	66.51 ± 1.70 *^a^*	19.83 ± 0.11 *^a^*	7.43 ± 0.25 *^a^*
		P	2138.28 ± 25.17 *^ab^*	409.71 ± 8.56 *^b^*	78.96 ± 2.82 *^a^*	75.71 ± 4.64 *^a^*	488.79 ± 12.87 *^a^*	93.91 ± 3.92 *^ab^*	88.86 ± 0.56 *^ab^*	63.47 ± 0.71 *^a^*	17.10 ± 1.30 *^a^*	7.15 ± 0.16 *^a^*
	SD	M	2195.29 ± 43.20 *^ab^*	297.84 ± 42.89 *^a^*	75.20 ± 6.68 *^a^*	81.04 ± 2.56 *^a^*	513.77 ± 6.54 *^ab^*	95.51 ± 2.62 *^ab^*	93.93 ± 2.61 *^b^*	69.19 ± 5.59 *^a^*	20.47 ± 1.75 *^a^*	8.24 ± 0.96 *^a^*
		I	2104.11 ± 13.60 *^a^*	375.77 ± 17.13 *^ab^*	70.40 ± 4.49 *^a^*	73.41 ± 0.86 *^a^*	491.11 ± 10.38 *^a^*	89.02 ± 1.96 *^a^*	88.74 ± 0.89 *^ab^*	63.08 ± 0.66 *^a^*	20.14 ± 0.18 *^a^*	7.24 ± 0.03 *^a^*
		T	2245.84 ± 28.76 *^b^*	405.81 ± 32.85 *^b^*	76.65 ± 5.52 *^a^*	75.52 ± 3.89 *^a^*	516.83 ± 2.38 *^ab^*	99.75 ± 0.56 *^b^*	91.29 ± 0.34 *^ab^*	67.26 ± 0.88 *^a^*	19.97 ± 0.36 *^a^*	7.67 ± 0.10 *^a^*
		P	2160.28 ± 35.23 *^ab^*	387.86 ± 32.76 *^ab^*	77.80 ± 5.53 *^a^*	73.17 ± 0.59 *^a^*	495.37 ± 0.07 *^ab^*	92.78 ± 3.44 *^ab^*	88.60 ± 4.47 *^ab^*	60.11 ± 0.80 *^a^*	18.68 ± 0.66 *^a^*	7.31 ± 0.36 *^a^*

J—juice; XE—XAD extract; FD—freeze drying; SD—spray drying_;_ M—maltodextrin; I—inulin; T—trehalose; P—palatinose; <LOD—below limit of detection (0.5 mg/100 g dm); ^A,B^—values followed by the same capital letter within a group (‘-’—no carrier) in a column are not statistically significantly different (*p* < 0.05) (Tukey’s HSD test); ^a,b^—values followed by the same lowercase letter within a group (juice) in a column are not statistically significantly different (*p* < 0.05) (Tukey’s HSD test); *^a,b^*—values followed by the same lowercase letter within a group (XAD extracts) in a column are not statistically significantly different (*p* < 0.05) (Tukey’s HSD test).

**Table 2:** The content of flavonols, flavan-3-ols, and iridoids in *Lonicera caerulea* L. powders (mg/100 g dry matter).

Sample	Drying Technique	Carrier	Flavonols												Flavan-3-Ols		Iridoids		
			Quercetin- 3-*O*-Rutinoside	Quercetin- 3-*O*-Glucoside	Quercetin- 3-*O*-Glucosyl-Xyloside	Isorhamnetin-3-*O*-Glucoside	Quercetin-*O*-Rhamnoside-*O*-Hexoside	Kaempferol- -3-*O*-Hexoside	Isorhamnetin-*O*-Hexosyl-*O*-Pentoside	Luteolin-7-*O*-Glucoside	Isorhamne-Tin 3-*O*-Rutinoside	Quercetin	Kaempferol-Rhamnosyl-Di-*O*-Glucoside	Quercetin-3-*O*-Vicianoside	(+)-Catechin	(−)-Epicatechin	Loganic Acid	7-Epi-Loganin	Pentosyl-Loganin
J	FD	-	209.43 ± 17.90 ^A^	37.77 ± 2.95 ^A^	34.04 ± 1.13 ^A^	9.39 ± 2.05 ^A^	<LOD	<LOD	11.68 ± 0.97 ^A^	5.47 ± 0.38 ^A^	<LOD	<LOD	<LOD	<LOD	83.17 ± 0.43 ^A^	46.63 ± 7.44 ^A^	13.96 ± 1.68 ^A^	12.42 ± 0.96 ^A^	10.09 ± 1.93 ^A^
XE	FD	-	2576.72 ± 48.70 ^B^	478.98 ± 23.76 ^B^	432.07 ± 23.45 ^B^	145.29 ± 2.63 ^B^	136.89 ± 11.17 ^A^	132.23 ± 10.12 ^A^	116.15 ± 12.36 ^B^	68.02 ± 4.49 ^B^	51.16 ± 2.17 ^A^	31.85 ± 5.12 ^A^	33.35 ± 2.62 ^A^	22.57 ± 2.85 ^A^	380.50 ± 16.11 ^B^	408.05 ± 10.59 ^B^	131.58 ± 3.91 ^B^	51.76 ± 1.86 ^A^	154.17 ± 4.35 ^C^
XE	SD	-	2561.42 ± 12.40 ^B^	469.03 ± 20.14 ^B^	403.15 ± 26.51 ^B^	159.24 ± 7.07 ^B^	222.50 ± 18.94 ^B^	150.57 ± 12.44 ^B^	117.96 ± 7.60 ^B^	65.26 ± 2.31 ^B^	46.70 ± 0.98 ^A^	29.42 ± 3.73 ^A^	30.58 ± 4.61 ^A^	42.75 ± 6.60 ^B^	350.28 ± 21.46 ^B^	371.92 ± 31.01 ^B^	121.21 ± 9.89 ^B^	103.07 ± 17.17 ^B^	107.90 ± 6.35 ^B^
J	FD	M	41.95 ± 1.20 ^a^	6.89 ± 0.44 ^ab^	7.92 ± 1.46 ^a^	<LOD	<LOD	<LOD	<LOD	<LOD	<LOD	<LOD	<LOD	<LOD	29.32 ± 2.17 ^a^	19.42 ± 0.55 ^a^	11.03 ± 1.74	4.66 ± 2.16 ^a^	<LOD
		I	39.05 ± 3.33 ^a^	6.22 ± 1.39 ^a^	6.23 ± 1.12 ^a^	<LOD	<LOD	<LOD	<LOD	<LOD	<LOD	<LOD	<LOD	<LOD	26.61 ± 0.96 ^a^	20.32 ± 3.59 ^a^	<LOD	4.20 ± 0.41 ^a^	<LOD
		T	42.13 ± 0.16 ^a^	7.61 ± 0.38 ^ab^	6.65 ± 0.42 ^a^	<LOD	<LOD	<LOD	<LOD	<LOD	<LOD	<LOD	<LOD	<LOD	27.15 ± 2.76 ^a^	<LOD	<LOD	<LOD	<LOD
		P	45.09 ± 6.10 ^a^	6.76 ± 0.78 ^ab^	6.77 ± 0.01 ^a^	<LOD	<LOD	<LOD	<LOD	<LOD	<LOD	<LOD	<LOD	<LOD	22.91 ± 2.62 ^a^	<LOD	<LOD	<LOD	<LOD
	SD	M	40.25 ± 1.35 ^a^	7.50 ± 0.70 ^ab^	6.76 ± 0.58 ^a^	<LOD	<LOD	<LOD	<LOD	<LOD	<LOD	<LOD	<LOD	<LOD	28.68 ± 1.92 ^a^	16.06 ± 3.22 ^a^	<LOD	<LOD	<LOD
		I	39.54 ± 5.84 ^a^	7.30 ± 0.46 ^ab^	7.17 ± 1.53 ^a^	<LOD	<LOD	<LOD	<LOD	<LOD	<LOD	<LOD	<LOD	<LOD	29.13 ± 2.76 ^a^	18.95 ± 0.37 ^a^	<LOD	<LOD	<LOD
		T	43.86 ± 0.26 ^a^	9.32 ± 0.89 ^b^	5.89 ± 1.49 ^a^	<LOD	<LOD	<LOD	<LOD	<LOD	<LOD	<LOD	<LOD	<LOD	28.61 ± 1.59 ^a^	<LOD	<LOD	<LOD	<LOD
		P	45.45 ± 3.13 ^a^	7.19 ± 0.19 ^ab^	5.84 ± 1.02 ^a^	<LOD	<LOD	<LOD	<LOD	<LOD	<LOD	<LOD	<LOD	<LOD	22.41 ± 2.57 ^a^	<LOD	<LOD	<LOD	<LOD
XE	FD	M	359.72 ± 8.15 *^a^*	64.31 ± 0.79 *^a^*	59.68 ± 2.19 *^a^*	16.21 ± 0.64 *^a^*	16.56 ± 3.35 *^a^*	16.85 ± 1.59 *^ab^*	15.72 ± 3.15 *^a^*	9.38 ± 0.55 *^a^*	<LOD	<LOD	<LOD	<LOD	72.46 ± 1.69 *^bc^*	80.84 ± 6.06 *^b^*	26.99 ± 1.15 *^a^*	14.49 ± 1.44 *^ab^*	25.71 ± 1.98 *^a^*
		I	347.39 ± 5.71 *^a^*	62.93 ± 0.84 *^a^*	57.55 ± 1.06 *^a^*	21.38 ± 3.23 *^a^*	16.63 ± 0.89 *^a^*	15.41 ± 0.46 *^a^*	17.98 ± 2.53 *^a^*	7.49 ± 1.26 *^a^*	<LOD	<LOD	<LOD	<LOD	76.43 ± 1.01 *^c^*	93.97 ± 7.73 *^b^*	25.26 ± 2.24 *^a^*	14.31 ± 0.15 *^ab^*	24.57 ± 3.49 *^a^*
		T	371.99 ± 4.32 *^a^*	67.65 ± 0.32 *^a^*	62.33 ± 0.05 *^a^*	21.35 ± 1.39 *^a^*	18.69 ± 0.21 *^ab^*	18.35 ± 1.30 *^ab^*	17.17 ± 1.38 *^a^*	7.68 ± 0.54 *^a^*	7.04 ± 0.81 *^a^*	<LOD	<LOD	<LOD	81.21 ± 5.51 *^c^*	82.82 ± 2.55 *^b^*	27.34 ± 4.75 *^a^*	15.08 ± 3.74 *^ab^*	24.20 ± 2.78 *^a^*
		P	353.86 ± 12.17 *^a^*	65.22 ± 6.62 *^a^*	53.51 ± 8.16 *^a^*	19.17 ± 2.70 *^a^*	20.08 ± 1.89 *^ab^*	22.43 ± 2.77 *^b^*	15.31 ± 1.58 *^a^*	7.20 ± 0.69 *^a^*	<LOD	<LOD	<LOD	<LOD	77.17 ± 0.27 *^c^*	77.40 ± 6.96 *^b^*	26.38 ± 2.11 *^a^*	9.32 ± 1.31 *^a^*	18.84 ± 0.03 *^a^*
	SD	M	377.66 ± 13.03 *^a^*	66.86 ± 6.13 *^a^*	61.29 ± 1.57 *^a^*	20.86 ± 0.88 *^a^*	<LOD	19.91 ± 1.92 *^ab^*	20.48 ± 1.47 *^a^*	9.65 ± 1.78 *^a^*	<LOD	<LOD	<LOD	<LOD	68.04 ± 5.22 *^a-c^*	93.52 ± 0.63 *^b^*	21.52 ± 1.49 *^a^*	19.55 ± 2.08 *^b^*	21.64 ± 1.02 *^a^*
		I	351.95 ± 8.17 *^a^*	65.13 ± 4.73 *^a^*	56.08 ± 0.96 *^a^*	20.18 ± 3.05 *^a^*	<LOD	19.25 ± 1.88 *^ab^*	16.96 ± 1.40 *^a^*	7.94 ± 0.47 *^a^*	8.02 ± 0.85 *^a^*	<LOD	<LOD	<LOD	58.66 ± 4.31 *^ab^*	91.96 ± 1.44 *^b^*	20.46 ± 2.01 *^a^*	14.84 ± 0.84 *^ab^*	22.26 ± 2.50 *^a^*
		T	369.00 ± 2.78 *^a^*	67.97 ± 0.14 *^a^*	61.54 ± 1.30 *^a^*	21.39 ± 3.66 *^a^*	18.40 ± 0.58 *^ab^*	16.00 ± 1.51 *^ab^*	18.21 ± 0.09 *^a^*	9.69 ± 1.19 *^a^*	<LOD	<LOD	<LOD	<LOD	55.07 ± 2.91 *^a^*	30.39 ± 1.58 *^a^*	60.42 ± 1.51 *^b^*	22.43 ± 1.52 *^c^*	<LOD
		P	350.51 ± 3.07 *^a^*	64.12 ± 4.38 *^a^*	60.28 ± 0.71 *^a^*	20.35 ± 3.73 *^a^*	22.96 ± 1.51 *^b^*	18.71 ± 1.29 *^ab^*	20.10 ± 0.29 *^a^*	9.74 ± 1.74 *^a^*	<LOD	<LOD	<LOD	<LOD	71.38 ± 5.05 *^bc^*	81.81 ± 9.21 *^b^*	22.78 ± 2.04 *^a^*	11.69 ± 0.83 *^a^*	24.78 ± 0.25 *^a^*

J—juice; XE—XAD extract; FD—freeze drying; SD—spray drying; M—maltodextrin; I—inulin; T—trehalose; P—palatinose; <LOD—below limit of detection (0.5 mg/100 g dm); ^A,B,C^—values followed by the same capital letter within a group (‘-’—no carrier) in a column are not statistically significantly different (*p* < 0.05) (Tukey’s HSD test); ^a,b^—values followed by the same lowercase letter within a group (juice) in a column are not statistically significantly different (*p* < 0.05) (Tukey’s HSD test); *^a,b,c^*—values followed by the same lowercase letter within a group (XAD extracts) in a column are not statistically significantly different (*p* < 0.05) (Tukey’s HSD test).

**Table 3:** Evaluation of the antioxidant, antiglycation, and anti-ageing potential of *Lonicera caerulea* L. powders.

			Antioxidant Assay		Antiglycation Assay				Anti-Ageing Assay	
			TEAC ABTS	FRAP	BSA–Glucose	BSA–Fructose	BSA–MGO	MGO–*L*-Arginine	Acetylcholinesterase	Butylcholinesterase
			(mmol Trolox/100 g dm)		(% of Fluorescent AGE Inhibition)				IC_50_ (mg/mL)	
J	FD	-	21.22 ± 0.15 ^b^	23.58 ± 0.73 ^b^	62.99 ± 0.70 ^d^	89.51 ± 0.21 ^d^	36.69 ± 2.89 ^c^	40.68 ± 4.72 ^e^	11.66 ± 0.99 ^a^	12.78 ± 1.08 ^a^
XE	FD	-	266.03 ± 2.89 ^e^	261.71 ± 8.44 ^d^	79.31 ± 0.93 ^e^	95.02 ± 0.06 ^d^	89.73 ± 0.03 ^f^	95.38 ± 0.47 ^g^	19.91 ± 1.69 ^b–e^	22.08 ± 1.87 ^a^
XE	SD	-	252.65 ± 1.95 ^d^	238.38 ± 5.96 ^c^	79.07 ± 0.76 ^e^	94.57 ± 0.22 ^d^	88.16 ± 0.43 ^f^	94.72 ± 0.23 ^g^	20.07 ± 1.71 ^b–e^	25.91 ± 2.20 ^a^
J	FD	M	3.83 ± 0.02 ^a^	4.71 ± 0.12 ^a^	50.27 ± 0.13 ^c^	66.18 ± 3.86 ^bc^	18.43 ± 0.44 ^ab^	−2.80 ± 0.97 ^a^	14.40 ± 1.22 ^ab^	24.06 ± 2.04 ^a^
		I	3.85 ± 0.10 ^a^	4.81 ± 0.08 ^a^	50.18 ± 0.98 ^c^	65.91 ± 0.73 ^bc^	15.31 ± 0.41 ^a^	4.41 ± 0.42 ^c^	21.17 ± 1.80 ^c–f^	22.42 ± 1.90 ^a^
		T	4.13 ± 0.19 ^a^	4.97 ± 0.08 ^a^	48.99 ± 0.34 ^bc^	64.89 ± 1.35 ^bc^	18.78 ± 0.46 ^ab^	10.05 ± 0.32 ^d^	13.92 ± 1.18 ^ab^	21.03 ± 1.78 ^a^
		P	3.92 ± 0.17 ^a^	4.92 ± 0.06 ^a^	45.27 ± 0.92 ^a^	54.26 ± 0.43 ^a^	18.73 ± 0.96 ^ab^	1.16 ± 0.15 ^a-c^	14.26 ± 1.21 ^ab^	23.30 ± 1.98 ^a^
	SD	M	3.25 ± 0.18 ^a^	4.07 ± 0.38 ^a^	50.68 ± 0.41 ^c^	63.17 ± 0.39 ^b^	18.02 ± 0.62 ^ab^	−2.33 ± 0.91 ^ab^	16.18 ± 1.37 ^a–c^	19.51 ± 1.67 ^a^
		I	4.05 ± 0.20 ^a^	5.05 ± 0.01 ^a^	46.74 ± 1.13 ^ab^	71.01 ± 0.64 ^c^	19.49 ± 0.74 ^ab^	2.20 ± 0.72 ^bc^	17.10 ± 1.45 ^a–d^	19.57 ± 1.61 ^a^
		T	4.06 ± 0.04 ^a^	5.12 ± 0.08 ^a^	49.16 ± 0.28 ^bc^	64.17 ± 0.48 ^bc^	21.50 ± 2.09 ^b^	2.24 ± 0.27 ^bc^	16.04 ± 1.36 ^a–c^	19.58 ± 1.66 ^a^
		P	3.71 ± 0.14 ^a^	4.82 ± 0.02 ^a^	46.89 ± 0.79 ^ab^	70.46 ± 5.64 ^c^	17.88 ± 1.71 ^ab^	3.47 ± 0.23 ^c^	16.41 ± 1.39 ^a–c^	20.08 ± 1.70 ^a^
XE	FD	M	23.99 ± 0.19 ^bc^	29.35 ± 0.64 ^b^	63.28 ± 0.81 ^d^	90.79 ± 0.18 ^d^	51.43 ± 0.63 ^e^	61.10 ± 0.06 ^f^	18.80 ± 1.60 ^b–e^	60.06 ± 5.10 ^b^
		I	24.42 ± 0.62 ^bc^	30.35 ± 0.91 ^b^	62.87 ± 0.71 ^d^	90.44 ± 0.03 ^d^	45.88 ± 0.14 ^d^	60.68 ± 0.64 ^f^	18.51 ± 1.57 ^b–d^	68.76 ± 5.83 ^b^
		T	26.01 ± 0.01 ^c^	32.98 ± 0.94 ^b^	62.97 ± 0.22 ^d^	90.62 ± 0.03 ^d^	46.86 ± 0.93 ^d^	60.27 ± 1.10 ^f^	17.21 ± 1.46 ^a–d^	61.57 ± 5.22 ^b^
		P	26.02 ± 0.37 ^c^	33.48 ± 0.26 ^b^	61.29 ± 0.25 ^d^	90.51 ± 0.19 ^d^	45.50 ± 1.06 ^d^	59.03 ± 0.08 ^f^	17.56 ± 1.49 ^a–d^	56.05 ± 4.76 ^b^
	SD	M	23.72 ± 0.51 ^bc^	28.13 ± 0.62 ^b^	63.27 ± 0.52 ^d^	94.57 ± 0.22 ^d^	49.43 ± 0.32 ^de^	59.67 ± 0.48 ^f^	25.21 ± 2.14 ^ef^	69.01 ± 5.85 ^b^
		I	24.23 ± 0.67 ^bc^	29.11 ± 1.63 ^b^	62.34 ± 0.27 ^d^	90.71 ± 0.04 ^d^	46.81 ± 0.45 ^d^	58.62 ± 0.28 ^f^	26.90 ± 2.28 ^f^	57.55 ± 4.88 ^b^
		T	25.21 ± 0.48 ^c^	31.43 ± 0.97 ^b^	63.55 ± 0.43 ^d^	90.57 ± 0.01 ^d^	46.51 ± 1.84 ^d^	60.64 ± 0.01 ^f^	23.03 ± 1.95 ^d–f^	63.55 ± 5.39 ^b^
		P	24.71 ± 0.47 ^c^	30.74 ± 1.61 ^b^	62.56 ± 0.25 ^d^	88.27 ± 3.07 ^d^	45.73 ± 0.01 ^d^	58.53 ± 0.82 ^f^	23.51 ± 1.99 ^d–f^	58.68 ± 4.98 ^b^

J—juice; XE—XAD extract; FD—freeze drying; SD—spray drying; M—maltodextrin; I—inulin; T—trehalose; P—palatinose; ^a–g^—values followed by the same letter in a column were not statistically significantly different (*p* < 0.05) (Tukey’s HSD test).

**Table 4:** Basic characterisation of *Lonicera caerulea* L. fruit and its fractions.

	Fruit	Juice	XAD Extract	Pomace
Dry matter (%)	14.03 ± 0.42 ^b^	12.93 ± 0.10 ^b^	6.02 ± 0.06 ^a^	38.29 ± 1.08 ^c^
Acidity (mg malic acid/100 g)	1.92 ± 0.01 ^c^	2.30 ± 0.07 ^d^	1.07 ± 0.02 ^a^	1.75 ± 0.01 ^b^
Vitamin C (mg ascorbic acid/100 g)	19.19 ± 0.19 ^c^	3.57 ± 0.16 ^b^	3.71 ± 0.03 ^b^	2.32 ± 0.02 ^a^
Ash (%)	0.38 ± 0.04 ^b^	0.43 ± 0.02 ^b^	0.07 ± 0.02 ^a^	1.05 ± 0.07 ^c^
Pectins (g/100 g)	1.01 ± 0.08 ^b^	ND	ND	0.23 ± 0.03 ^a^

ND—not detected; ^a–d^—different letters within a row indicate a statistically significant difference (*p* < 0.05) (Tukey’s HSD test).

## Data Availability

The original contributions presented in the study are included in the article. The raw data supporting the conclusions of this article will be made available by the authors on request.
